# What Can We Learn from “Not Much More than *g*”?

**DOI:** 10.3390/jintelligence5010008

**Published:** 2017-02-25

**Authors:** Kevin Murphy

**Affiliations:** Kemmy Business School, University of Limerick, Limerick V94 T9PX, Ireland; krm10@me.com

**Keywords:** general cognitive ability, second stratum abilities, specific ability, complex problem solving

## Abstract

A series of papers showing that measures of general cognitive ability predicted performance on the job and in training and that measures of specific cognitive abilities rarely made an incremental contribution to prediction led to a premature decline in research on the roles of specific abilities in the workplace. Lessons learned from this research include the importance of choosing the right general cognitive measures and variables, the relative roles of prediction vs. understanding and the need for a wide range of criteria when evaluating the contribution of specific skills such as complex problem solving. In particular, research published since the “not much more than *g*” era suggests that distinguishing between fluid and crystallized intelligence is important for understanding the development and the contribution of complex problem solving.

## 1. Introduction

Many jobs require incumbents to attain skills in solving problems that are nonroutine and dynamic, and that require both the acquisition and application of new knowledge. These Complex Problem Solving (CPS) skills are likely to become an increasingly important determinant of job performance and occupational success [1], There is a long history of research on the cognitive underpinnings of job performance, and this research suggests that identifying a distinct role for specific skills such as complex problem solving is important, but that it is made substantially more difficult as a result of the hierarchical structure of human cognitive abilities.

A series of widely cited papers examining the role of specific vs. general cognitive abilities in predicting performance in jobs and in training suggest that the prediction of performance in complex tasks often involves “not much more than *g*”, and that more specific abilities rarely make a meaningful contribution to predicting external criteria over and above the contribution of more general abilities [2,3,4,5,6,7,8]. That is, once general cognitive factors have been taken into account, it is rare to find that adding measures of job-specific abilities leads to a substantial change in the ability to predict performance or effectiveness on most jobs [3,4,5,6,7,8,9]. Indeed, there is considerable evidence that the criterion-related validity of cognitive tests, including those that vary widely in content, does not vary greatly across jobs [3,4,5,7,10,11]. This pattern of results is often taken as evidence that specific abilities and skills make only a minor contribution to the prediction of performance in complex tasks and jobs once general cognitive abilities are taken into account. Hunter [11] has argued that the evidence supports an even stronger version of this argument, claiming that it is general ability and not specific abilities that predict performance.

## 2. The Hierarchical Structure of Cognitive Abilities

Cognitive tests of virtually all sorts show a consistent pattern of positive correlations among tests and between tests and criteria, i.e., positive manifold [3,12,13,14]. This pattern is by no means limited to the domain of cognitive tests; Ree, Carretta and Teachout [15] suggest that it generalizes across a number of domains. Positive manifold has two implications that are important for understanding the relative roles of general vs. specific cognitive factors in explaining outcomes such as task or job performance [16,17,18]. First, positive manifold guarantees that the scores on all tests and composites of tests will be positively correlated with the composite of tests that accounts for the largest possible percentage of variance in performance across all tests, i.e., the first principal component of any set of reliable tests of information processing. If the set of tests is a reasonably broad one, this first principal component is often interpreted as a measure of the most general cognitive factor, “*g*” [17]. Second, scores on any measure of a specific cognitive ability or skill will reflect variance that is unique to that particular ability or skill and variance that can be explained in terms of *g*.

Jackson, Putka and Teoh [19] pointed out that when the data are multifaceted (e.g., tests differ not only in content but in terms of their source (e.g., self-ratings vs. ratings from others) or their mode of measurement), the determination of general factors can be more complex, and that the interpretation of scores on an optimal composite of all tests as indicating a general factor cannot always be assumed. The likelihood of finding a strong general factor underlying a set of cognitive tests can also depend on the methods used to analyze test data [20]. Nevertheless, it is clear that scores on measures of any specific cognitive ability or skill will reflect both general and specific factors. Indeed, current research suggests that complex problem-solving skills are strongly related to general cognitive factors, even though there are both conceptual and empirical distinctions between complex problem solving and general cognitive ability [21].

The consistent presence of positive manifold virtually guarantees that hierarchically organized models, with the most specific abilities and skills forming the lowest stratum and a small set of general abilities forming the top stratum, must emerge. The most widely supported theories of human cognitive ability take on this hierarchical structure [13,22,23,24]. The consistent success of measures of general cognitive ability in predicting work-related criteria, and the persistent failure of measures of more specific abilities to add substantially to the predictive power of models that already contain general cognitive factors [3,8,10,25,26,27] had the effect of discouraging research on specific ability measures in the workplace for many years. Research on this topic is just starting to re-emerge [28,29,30,31].

Measures of CPS skills are highly correlated with measures of general cognitive ability [32]. However, several authors have argued that these skills are both empirically and conceptually distinct from general cognitive ability [21,32,33,34,35,36]. In particular, CPS represents a particular set of skills in acquiring and using information to solve problems that an individual has not previously encountered and that cannot be addressed using familiar information or routines. CPS clearly draws upon more general abilities in information processing, but it is a particular skill that can be acquired and improved over time. Nevertheless, the strong relationship between CPS and “g” might lead some researchers to conclude that CPS is unlikely to make a worthwhile contribution to predicting and understanding job performance and occupational success.

## 3. Lessons Learned

A review of the development and effects of research on the role of general vs. specific abilities as predictors of success in jobs and in training suggests a number of important lessons for current studies that are designed to examine the relationship between general cognitive abilities and complex problem-solving skills. These can be grouped under three broad headings: (1) which general abilities; (2) why ask the question; and (3) what are the criteria?

### 3.1. Which General Abilities?

Campbell [37] notes that there are two types of general factors, those that have direct explanatory power as causes of the behaviors of interest and those that are artifacts, caused by the additive effects of the more specific variables they represent. That is, we can either think of general mental ability as a latent variable that is the cause of performance or as a formative variable that is the outcome of combining a number of inter-related specific abilities [38,39]. This distinction reflects some of the earliest attempts to define cognitive ability, with Spearman [40] arguing that *g is* intelligence, and that it is *g* that causes people to perform well or poorly on a wide range of tasks, whereas Thurstone [41] argued that *g* is a composite of more interpretable group factors, and that it summarizes rather than explains performance [42].

Until recently, virtually all of the studies that have examined the roles of general versus specific mental abilities on performance in the workplace have focused on the type of general factor that is best approximated by the first principal component emerging when a set of cognitive measures is analyzed. As noted earlier, this type of measure is widely interpreted as a good approximation of Spearman’s *g* or of the single general factor that resides at the top of many hierarchical models. This general factor is optimal as a statistical summary of many different abilities, but it is also difficult to interpret in any substantive terms [37,39,43].

On the other hand, most models of cognitive ability recognize the existence and importance of factors at the second-highest stratum of hierarchical models that are quite general in nature but are also more substantively meaningful than the highest-order factor *g*, the most obvious being fluid and crystallized intelligence (*gf* and *gc*, respectively). These second-stratum factors have considerable generality, albeit being less inclusive than *g*, and there are several advantages of working with such second-stratum factors rather than with *g* in both research and practice. First, there is much more consensus about the meaning and the content associated with factors such as fluid and crystallized intelligence, short-term memory, visual processing and processing speed than there is about *g*. There is evidence to link CPS with fluid intelligence [34,35]; the conceptual relevance of this aspect of general intelligence to CPS seems substantially clearer than the general *g*-CPS link. Because the basis for CPS skills is the acquisition and use of new information, assessing the store of current information and knowledge (i.e., crystallized intelligence) may contribute less to understanding CPS skills and more to targeted assessments of relevant aspects of cognitive ability. Second, recent research suggests there are practical advantages to using measures of second-stratum factors rather than *g* to predict workplace criteria, including potential increases in predictive ability and decreases in the adverse impact of cognitive tests on the employment opportunities of members of racial and ethnic minority groups [28,29,30], and it is likely that second-stratum measures, particularly assessments of fluid intelligence, will supplant measures of *g* in at least some workplace settings. Studies by Ree and his colleagues and other researchers that appear to show decisive advantages of *g* measures over measures of more specific abilities failed to consider the types of second-stratum abilities that are beginning to be seen as more useful than more global measures of general cognitive ability for predicting and explaining job performance. Thus, understanding the relationships between complex problem solving and these second-stratum factors is more likely to shed light on precisely what determines the acquisition and development of skill in solving complex problems than would be gained by studying complex problem solving in the light of *g*.

### 3.2. Why Ask the Question?

Another way to understand the question of which general abilities should be the focus of research on complex problem solving is to consider why different people might ask this question. Campbell [36] notes that different types of general factors might be optimal for different purposes. In particular, the most general measures of cognitive ability have an undeniable advantage in terms of their combined predictive power, generality and simplicity. If you want to be reasonably certain of predicting performance in virtually any job, measures of the most general cognitive abilities will almost certainly be your best choice. If predictive efficiency is the goal, it will be hard to consistently beat *g*.

If the goal is understanding rather than prediction, *g* may be a uniquely bad choice as a tool for shedding light on the meaning, determinants, and contributions of measures of complex problem solving. In contrast to second-stratum abilities, *g* is poorly defined and poorly understood. In particular, most widely used measures of general cognitive ability (e.g., tests such as the Wonderlic, scores on the first principal component of multi-test batteries) confound fluid and crystallized intelligence. Thus, even if the incremental role of complex problem-solving skills in relationship to measures of *g* can be firmly established, the value of this knowledge for understanding complex problem solving may be minimal.

### 3.3. What Are the Criteria?

Finally, the choice of criteria is important when evaluating the role of complex problem solving versus various general mental ability measures. When the criteria are themselves quite general and poorly differentiated (e.g., overall job performance), the most general ability measures may have an advantage [37]. On the other hand, Rojon, McDowall and Saunders [44] have shown that it is possible to achieve higher levels of validity when criteria are specific and the predictor is sensibly matched with particular criteria.

When the criteria are broad and general, it can be challenging to show that measures of complex problem solving make an incremental contribution to prediction, see [33], however. It can be argued that studies of the contribution of specific cognitive abilities to the prediction of success on the job or in training prematurely cut off debate on this question because they almost always relied on broad and general criteria. If we are going to seriously examine the incremental contribution of measures of complex problem solving, it will be important to employ a range of criteria, including specific measures where a conceptual argument can be made for the incremental contribution of measures of this specific skill.

## 4. Summary

For several decades, the question of whether measures of specific cognitive ability contributed anything meaningful to the prediction of performance on the job or performance in training once measures of general mental ability were taken into account appeared to be settled, and a consensus developed that there was little value in using specific ability measures in contexts where more general measures were available. It now appears that this consensus was premature, and that measures of specific abilities can make important contributions even if general measures are taken into account.

The debate over the role of general vs. specific abilities in predicting performance on the job or in training suggests that it is important to first think carefully about what type of general measure to use, and more importantly, why use that general measure. If the goal is to do a good job in prediction, while minimizing the complexity of the predictive model, it makes sense to use the most general measures available. If the goal is to understand the meaning and determinants of complex problem solving, there is a strong argument for using measures that are somewhat less general (e.g., *gf*) but easier to understand.

Finally, the choice of criteria is very important in drawing conclusions about the incremental value of measures of specific skills, such as complex problem solving. The more general the criteria, the more valuable a measure of the most general ability and the more difficult it will be to demonstrate incremental value for measures of specific abilities and skills. The more specific the criterion, and the stronger its conceptual links to complex problem solving, the easier it should be to demonstrate that specific measures make an incremental contribution.
